# Clinical Peculiarities in a Cohort of Patients with Wolfram Syndrome 1

**DOI:** 10.3390/ijerph19010520

**Published:** 2022-01-04

**Authors:** Giuseppina Salzano, Luciana Rigoli, Mariella Valenzise, Roberto Chimenz, Stefano Passanisi, Fortunato Lombardo

**Affiliations:** 1 Department of Human Pathology in Adult and Developmental Age “Gaetano Barresi”, University of Messina, Via Consolare Valeria 1, 98125 Messina, Italy; lrigoli@unime.it (L.R.); mvalenzise@unime.it (M.V.); stepassanisi@unime.it (S.P.); fortunato.lombardo@unime.it (F.L.); 2 Unit of Pediatric Nephrology and Dialysis, Department of Human Pathology in Adult and Developmental Age “Gaetano Barresi”, University of Messina, 98125 Messina, Italy; rchimenz@unime.it

**Keywords:** chronic renal failure, deafness, diabetes mellitus, DIDMOAD, genetics, optic atrophy, therapy, wolframin

## Abstract

Wolfram syndrome 1 is a rare, autosomal recessive, neurodegenerative, progressive disorder. Insulin-dependent, non-autoimmune diabetes mellitus and bilateral progressive optic atrophy are both sensitive and specific criteria for clinical diagnosis. The leading cause of death is central respiratory failure resulting from brainstem atrophy. We describe the clinical features of fourteen patients from seven different families followed in our Diabetes Center. The mean age at Wolfram syndrome 1 diagnosis was 12.4 years. Diabetes mellitus was the first clinical manifestation, in all patients. Sensorineural hearing impairment and central diabetes insipidus were present in 85.7% of patients. Other endocrine findings included hypogonadotropic hypogonadism (7.1%), hypergonadotropic hypogonadism (7.1%), and Hashimoto’s thyroiditis (21.4%). Neuropsychiatric disorders were detected in 35.7% of patients, and urogenital tract abnormalities were present in 21.4%. Finally, heart diseases were found in 14.2% of patients. Eight patients (57.1%) died at the mean age of 27.3 years. The most common cause of death was respiratory failure which occurred in six patients. The remaining two died due to end-stage renal failure and myocardial infarction. Our data are superimposable with those reported in the literature in terms of mean age of onset, the clinical course of the disease, and causes of death. The frequency of deafness and diabetes insipidus was higher in our patients. The incidence of urogenital diseases was lower although it led to the death of one patient. Long-term follow-up studies including large patient cohorts are necessary to establish potential genotype-phenotype correlation in order to personalize the most suitable clinical approach for each patient.

## 1. Introduction

According to the draft International Classification of Disease (ICD-11), Wolfram syndrome (WS) is defined as a rare, specified form of diabetes mellitus (DM) caused by other genetic syndromes. WS is an autosomal, recessive, progressive neurodegenerative disease characterized by early-onset non-autoimmune insulin-dependent DM and bilateral optic neuropathy (OA), which are the key clinical criteria used to make a diagnosis [1,2]. It was first reported by Wolfram and Wagener in 1938. Epidemiological studies on WS are scarce. To date, the estimated prevalence varies from 1:100,000 to 1:805,000 depending on different ethnic groups [3,4,5,6,7]. Two different subtypes of WS are currently described, namely WS1 and WS2, which are associated with two different disease-genes, *WFS1* and *CISD2,* respectively [8]. The *WFS1* gene maps to chromosome 4p16.1, and consists of eight exons encoding wolframin, which is a pleiotropic transmembrane protein located in the endoplasmic reticulum (ER) with 890 amino acid residues and an apparent molecular mass of about 100 kDa [9]. Current evidence suggests that wolframin plays a crucial role in regulating ER-mitochondria homeostasis. More than 250 different variants have been identified in the *WFS1* gene. These variants include missense, nonsense, frameshift mutations, and also small deletions, insertions, and duplications [10]. Pathogenic *WFS1* variants cause ER stress in pancreatic β-cells, neurons, retinal ganglion cells, and oligodendrocytes, resulting in the dysfunction and degeneration of affected tissues [10]. *CISD2* maps to chromosome 4q22-q24 and is expressed in several tissues, including the brain and pancreas [11]. It encodes a small endoplasmic reticulum (ER) intermembrane protein of 135 amino acids, called ERIS, which is located in mitochondria-associated ER membranes and is involved in the regulation of glucose and calcium homeostasis [12].

WS1 is also known as DIDMOAD due to its cardinal clinical features, which are diabetes insipidus (DI), DM, OA, and deafness [13]. Other clinical manifestations include neurological progressive disabilities, structural and functional urinary tract abnormalities, psychiatric disorders, and other endocrine system dysfunctions [14]. To date, the management of patients with WS1 is only aimed at treating all disease-related clinical manifestations. There is currently no treatment to delay, halt, or reverse the progression of WS1 [15]. Consequently, the prognosis is poor as most patients die prematurely with severe neurological disabilities, such as bulbar dysfunction and organic brain syndrome [6,16]. Death usually occurs in the third to fourth decades of life. The leading cause of death is respiratory failure as a result of brain stem atrophy [17].

The aim of this study is to describe the clinical presentation in a relatively large cohort of Caucasian patients with WS1, who were born and live(d) in Sicily, a southern region of Italy.

## 2. Material and Methods

A retrospective analysis was conducted to identify all patients diagnosed with WS1 between January 1998 and July 2021 and followed up at our tertiary Diabetes Centre, which is the only recognized reference Center in the Messina district for diagnosis, treatment, and follow-up of youth-onset diabetes. Diagnosis of WS1 was made through genetic testing, which was performed by analyzing eight exons and the exon–intron sequences of the *WFS* gene. All clinical data of our study cohort were gathered by reviewing clinical records starting from the time of diagnosis of insulin-dependent DM. The study was conducted in accordance with the Helsinki Declaration, good clinical practice, and all applicable laws and regulations. Every patient, if of age, or at least one parent if underage, gave their written informed consent before the start of study procedures. The study was exempt from ethical committee approval since it was confined to anonymized and unidentifiable data routinely collected at our Diabetes Centre.

### 2.1. Genetic Analysis

Genetic testing for WS1 was performed at the Genetic Unit of our University Hospital in patients presenting with basic clinical criteria contributing to WS1 diagnosis, i.e., the coexistence of non-autoimmune insulin dependent DM, and OA [1]. After genomic DNA was extracted, the *WFS* gene was amplified using the Gene Amp PCR System 9700 (Perkin Elmer, Foster City, CA, USA) with an initial denaturation step at 94 °C for 15 min and then 30 cycles at 94 °C for 30 s, 60 °C for 30 s, 72 °C for 45 s, followed by 7 min of final extension at 72 °C. Amplicons were sequenced for both sense and antisense strands using an automated fluorescent sequencing method (Big Dye Terminator kit v1.1, Applied Biosystems). The samples were analyzed on an ABI Prism sequencing apparatus 3730 (Applied Biosystem, Foster City, CA, USA). Sequencing of both DNA strands of three independent PCR products was performed to validate all variations. The sequence variants were considered mutations if they caused a non-conservative amino acid change or were absent in 300 ethnically matched control chromosomes or affected phylogenetically conserved residues. Other DNA variations that did not fulfill these criteria were considered polymorphisms.

### 2.2. Bioinformatics

All the identified WFS1 variants were checked utilizing HGMD (http://www.hgmd.cf.ac.uk/ac/index.php; accessed on 25 October 2021), Exac (http://exac.broadinstitute.org/; accessed on 25 October 2021), EVS (http://evs.gs.washington.edu/EVS/; accessed on 25 October 2021), dbSNP (https://www.ncbi.nlm.nih.gov/projects/SNP/; accessed on 25 October 2021), and Varsome (https://varsome.com/; accessed on 25 October 2021). The potential pathogenic role of mutations on WFS1 functionality was defined by several computational analyses, such as Mutation Taster, Sift, and Polyhen2, which classified variants as “disease causing”, “damaging”, and “probably damaging”, respectively.

### 2.3. Study Population

During the study period, a total of twenty-one patients underwent genetic investigations. Of these, fourteen patients (66.7% of the cases) were diagnosed as WS1 and represented our study population. A significant portion of the material has already been published in a previous paper of our study group [13]. However, since that publication date, other WS diagnoses have been made and the clinical course of previously described patients has been updated in this manuscript. All patients had been born in the Messina district. Of these, eight patients died at the mean age of 27.3 years. At the time of this study, the mean age of the remaining living subjects was 22.5 (ranging from 12–30) years. All the patients were, or have been followed, at our Diabetes Centre since the onset of insulin-dependent, non-autoimmune diabetes. The mean age at diagnosis of WS1 was 12.7 ± 3.4 years (5–19 years).

Seven patients of our study population descend from a five-generation WS1 pedigree: four siblings (two brothers and two sisters) were found in the 5th generation, and three siblings (two sisters and one brother) in the 4th generation of the pedigree. The parents of both 4th and 5th generation WS1 patients were consanguineous. The remaining seven WS1 patients of the study group, which also included two sisters, were from unrelated families and were born to healthy non-consanguineous parents. Nine patients, including those belonging to the same pedigree, came from two small towns in the Nebrodi Mountains, while the other five patients lived in different areas of the Messina district.

Most of our patients (eleven out of fourteen of the WS1 patients analyzed) had the same *WFS* gene variants occurring in exon 8b. It was a Y454_L459del_fsX454 homozygous frameshift/truncation mutation causing a 16 base-pair deletion (c1362_1377del16) localized in the CD3 cytoplasmic domain. Missense mutations were detected in two patients. One of these was a K178_A179del/R558H compound heterozygous. The K178_A179del mutation was a six c532_537deletion localized in the N-terminal region. R558H mutation (c1673G>A), a missense mutation, was found in the CD4 cytoplasmic domain of the wolframin. Another patient was compound heterozygous for the S443I mutation and c.712+16G>A. A S443I missense mutation was found at np1328 (AGC>ATC) in exon 8, leading to a substitution of serine to isoleucine. It was situated in the TM4 transmembrane region. c.712+16G>A was situated at intron 6. Finally, the remaining patient was compound heterozygous for a L410del-fsX441/F516del. L410del-fsX441 was a four-nucleotide deletion (c.1230delCTCT) (frameshift/truncation mutation) localized in the TM3-TM4 transmembrane regions. A F516del mutation (c.1246delTTC) was found in the ER3 endoplasmic reticulum region of wolframin (Table 1).

Results of patient genetic analyses were supported by the results of their parents’ molecular investigations. All the detected mutations had been previously described.

## 3. Results

The main clinical features of our study population are summarized in Table 2.

### 3.1. Diabetes Mellitus

The mean age at DM onset was 5.9 ± 4.3 years. Diabetic ketoacidosis, defined by blood glucose levels > 250 mg/dL, pH < 7.3, and serum bicarbonate levels < 15 mEq/L, occurred in 14.3% of our study population. In all cases, DM appeared earliest among other clinical manifestations of WS1, and was diagnosed in the first decade in nearly all patients (92.9% of the study cohort). The mean glycated hemoglobin (HbA1c) value at the time of diagnosis was 10.2% (88 mmol/mol). Diabetes-specific autoimmunity, assessed by the titration of at least two autoantibodies against glutamic acid decarboxylase 65 autoantibodies, tyrosine phosphatase-like insulinoma antigen 2, insulin, and β-cell-specific zinc transporter 8 autoantibodies, was absent in all cases. Type 1 diabetes predisposing human leukocyte antigen (HLA) alleles (i.e., HLA-DRB1, HLA-DQA1, and HLA-DQB1) were found in 14.3% of subjects. During the follow-up period, the insulin daily dose requirement was persistently < 1 IU/kg for all patients, and the mean HbA1c levels were 6.9% (52 mmol/mol). No hospitalizations due to acute metabolic complications (i.e., diabetic ketoacidosis, hyperosmolar hyperglycemic nonketotic coma, and severe hypoglycemia) occurred. No microvascular complications of diabetes were detected.

### 3.2. Other Clinical Findings

OA was diagnosed in our patients at the mean age of 12.7 ± 3.4 years and mainly appeared in the second decade of life. The mean time between diagnosis of DM and OA was 6.8 years. Deafness was present in 85.7% of patients and was diagnosed prior to OA in half of the cases. DI was found in twelve patients (85.7%) and diagnosed according to the following clinical findings: polyuria, polydipsia, urine osmolality of <300 mOsm per kilogram of water, or urine specific gravity of <1010 in a 24-h urine sample without glycosuria and ketonuria. The mean age at DI diagnosis was 14.1 ± 3.8 years. Renal out-flow tract abnormalities were detected in three patients, and in one case were related to the appearance of chronic renal failure. Neuropsychiatric disorders, including depression and anxiety, were diagnosed in 35.7% of our study population. Endocrine system abnormalities were revealed in five patients and, in particular, three subjects were affected by Hashimoto’s thyroiditis, one patient had hypergonadotropic hypogonadism, and another one was diagnosed with hypogonadotropic hypogonadism. Both patients with hypogonadism were males. Other less common clinical features included bilateral cataract and heart diseases, including ventricular septal disease and secundum atrial septal defect with concomitant valvulopathy. Both these clinical findings were found in 14.3% of our study population.

As mentioned above, eight patients died. The mean age of death in our study population was 28.3 years (19–32 years). The most common cause of death was respiratory failure which occurred in six patients. The other two patients died due to end-stage renal failure and myocardial infarction.

## 4. Discussion

WS1 is a rare but insidious neurodegenerative disease that should be suspected in patients with non-autoimmune DM and ophthalmological disorders. As confirmed by our data, DM is the first clinical manifestation to appear. Treatment is based on the basal-bolus insulin regimen. Compared with type 1 diabetes, DM in WS1 patients is characterized by a lower, daily insulin requirement and a milder clinical course than type 1 diabetes. Both acute and chronic complications are rare [18]. HLA predisposing to type 1 diabetes cannot be considered an exclusion criterion to suspect WS1.

OA with resultant loss of visual acuity rapidly leads to blindness and usually occurs in the second decade of life. Ophthalmological diagnostic tests such as color vision testing, fundoscopy, visual field, optical coherence tomography scan, and retinal thinning are useful to evaluate disease progression [19]. Some authors have hypothesized potential benefits from the use of idebenone or docosahaexanoic acid to slow down the progression of OA [20,21], but no proven active treatment is available to date. Other eye disorders may be present in WS1 patients. Among these, bilateral cataract is the most common and this developed in two patients of our study cohort. Of note, Berry et al. reported a family with isolated congenital cataracts due to a missense mutation in the *WFS* gene [22].

Excluding the DM and OA present in all our patients, deafness and DI were the other most common clinical manifestations in our study population occurring in 85.7% and 92.9%, respectively. Our findings are inconsistent with those reported by De Heredia et al. in their analysis of data from 412 patients. The authors showed that 48.2% of WS1 patients had deafness, and 37.8% had DI [23]. Other studies showed that sensorineural hearing loss involves about 70% of WS1 patients [24,25]. Higher frequencies of deafness and DI in our patients may be explained by the longer follow-up period of our study that allowed us to observe the appearance of several clinical findings. Moreover, the likelihood that the genetic variants presented by most of our patients may be more frequently related to the appearance of deafness and DI than the genotype of unrelated patients described in other larger studies should not be overlooked. Hearing impairment usually manifests in the second decade and may sometimes appear prior to ophthalmological defects, as demonstrated in our experience. Hearing loss affects the high frequencies first and progresses relatively slowly [6,26]. Regular monitoring is suggested for appropriate treatment, and hearing aids and cochlear implants are currently the most suitable therapeutic tools for these patients [27]. Dominant mutations in the *WFS* gene have been described as a common cause of low-frequency sensorineural hearing loss in patients who do not develop other cardinal symptoms of WS1 [28,29]. DI occurs mostly in the second to third decade of life. It is usually responsive to treatment with desmopressin [30]. Other endocrine disorders are not rare in WS1 patients, as demonstrated in our study. Primary and secondary hypogonadism may be present in male patients, while menstrual abnormalities and infertility have been reported in female patients [8,31]. However, cases of pregnant patients have also been described [32]. Finally, additional endocrine diseases, such as hypothyroidism and growth retardation, have been observed in WS1 patients [33].

Renal out-flow tract abnormalities were present in 21.4% of our patients. Neurogenic bladder associated with hydroureteronephrosis, urinary incontinence, and recurrent urinary tract infections is not uncommon clinical findings [34,35]. Yearly assessment of renal function, measurement of postvoid residual urine volume by ultrasound, a renal ultrasound, and urodynamic testing are strongly recommended in WS1 patients [1]. Urological manifestations may significantly affect quality of life and may be complicated by the occurrence of chronic renal failure, as reported in one patient of our study population.

Multiple neurological abnormalities may be detected in WS1 patients, including coordination deficit ataxia, central apnea, dysarthria, aphasia, autonomic neuropathy, peripheral neuropathy, alterations in sleep/wake rhythm, headache, and seizures. Magnetic resonance imaging may reveal cortical atrophy [1,31]. Anxiety and depression are the most common among neuropsychiatric disorders [36,37]. A significant increase in suicidal behavior has also been reported [38]. Conversely, cognitive function does not seem to be altered in patients with WS1, especially in children and adolescents [39].

Several studies have been conducted to establish a genotype-phenotype correlation [4,14,17,18,23]. It has been reported that compound heterozygous patients have a higher risk of psychiatric disorders, DM, and deafness [23]. Some studies have shown that patients who are homozygous or compound heterozygous for two inactivating mutations had an earlier onset of the disease [18]. Furthermore, compound heterozygosity for missense mutations may lead to a mild phenotype [17,23]. Finally, patients who have complete loss-of-function mutations appear to develop DM at an earlier age than those who have partial loss-of-function mutations [4]. However, it is currently hard to correlate the genetic variant with the clinical course of the disease due to the molecular complexity of WFS1, the wide clinical heterogeneity, and the small size of patient cohorts available [2].

Although the management of WS1 is still supportive, numerous studies are currently ongoing to evaluate the effectiveness of new therapeutic strategies. Chemical chaperones, such as 4-phenylbutyric acid and tauroursodeoxycholic acid, seem to be the most promising among novel treatments [15]. They are small compounds that are known to mitigate ER stress by rescuing or stabilizing the native conformation of mutant *WFS* proteins, and also slow down the neurodegeneration process [40]. Another potential therapeutic approach is based on the use of ER calcium stabilizers. Dantrolene sodium, a Food and Drug Administration (FDA)-approved drug for malignant hyperthermia and muscle spasm, has been demonstrated to suppress cell death and dysfunction in neuronal and β-cell animal models of WS1 syndrome, as well as in induced pluripotent stem cell models of this disease [20]. Valproate acid is also being investigated as a novel therapy for WS1 patients (Clinical Trial Number: NCT03717909) and has been shown to confer protection against cell death in WS1 [41]. Recently, some studies have reported that GLP-1 receptor agonists appear to be effective in alleviating cellular stress and improving β-cell function in mouse models of WS1 syndrome [42,43]. Finally, the efforts of researchers are currently aimed at identifying potential definitive cures for WS1 by exploiting innovative approaches such as gene therapy or regenerative medicine [44].

## 5. Conclusions

WS1 is a rare neurodegenerative disease that is still characterized by complex heterogeneous clinical expressivity, poor quality of life, and shortened lifespan. A multidisciplinary approach is mandatory starting from the time of diagnosis to promptly identify and treat also less common clinical manifestations, such as endocrine system abnormalities and cardiac diseases. Finally, long-term follow-up studies including large patient cohorts are required to assess potential genotype-phenotype correlations.

## Figures and Tables

**Table 1:** Mutations and genotype of WS1 patients.

N	Gene	Exon	Type of Mutation	Nucleotide Change	Protein Effect	Protein Domain	Zygosity	Known/Novel
1	WFS1	8b	Frameshift/truncation	c1362_1377del16	Y454_L459del_fsX454	CD3	Homozygote	Known
2	WFS1	8b	Frameshift/truncation	c1362_1377del16	Y454_L459del_fsX454	CD3	Homozygote	Known
3	WFS1	8b	Frameshift/truncation	c1362_1377del16	Y454_L459del_fsX454	CD3	Homozygote	Known
4	WFS1	8b	Frameshift/truncation	c1362_1377del16	Y454_L459del_fsX454	CD3	Homozygote	Known
5	WFS1	8b	Frameshift/truncation	c1362_1377del16	Y454_L459del_fsX454	CD3	Homozygote	Known
6	WFS1	8b	Frameshift/truncation	c1362_1377del16	Y454_L459del_fsX454	CD3	Homozygote	Known
7	WFS1	8b	Frameshift/truncation	c1362_1377del16	Y454_L459del_fsX454	CD3	Homozygote	Known
8	WFS1	8b	Frameshift/truncation	c1362_1377del16	Y454_L459del_fsX454	CD3	Homozygote	Known
9	WFS1	8b	Frameshift/truncation	c1362_1377del16	Y454_L459del_fsX454	CD3	Homozygote	Known
10	WFS1	5/8c	Deletion; missense	c532_537del6; c1673G>A	K178_A179del; R558H	CD1; CD4	Compound heterozygote	Known
11	WFS1	8b/IVS6	Missense; splice	c.1328G>T; c.712+16G>A	S443I; –	TM4; -	Compound heterozygote	Known
12	WFS1	8b/8c	Frameshift/truncation; deletion	c1230delCTCT; c.1246delTTC	L410del-fsX441; 516del	TM3-TM4; ER3	Heterozygote	Known
13	WFS1	8b	Frameshift/truncation	c1362_1377del16	Y454_L459del_fsX454	CD3	Homozygote	Known
14	WFS1	8b	Frameshift/truncation	c1362_1377del16	Y454_L459del_fsX454	CD3	Homozygote	Known

**Table 2:** Clinical features of our study cohort.

**Number of WS** **1 Patients.**	**14**
Sex	
Female	6 (42.9%)
Male	8 (57.1%)
Age at WS1 diagnosis (years)	12.7 ± 3.4
**Diabetes mellitus**	
Number of patients	14 (100%)
Mean age at diagnosis (years)	5.9 ± 4.3
Age range at diagnosis (min–max years)	1–18
Diabetic ketoacidosis at onset	2 (14.3%)
Glycated hemoglobin at diagnosis (%)	10.3 ± 2.1
**Optic atrophy**	
Number of patients	14 (100%)
Mean age at diagnosis (years)	12.7 ± 3.4
Age range at diagnosis (min–max years)	6–19
**Diabetes insipidus**	
Number of patients	12 (85.7%)
Mean age at diagnosis (years)	14.2 ± 3.9
Age range at diagnosis (min–max years)	10–22
**Deafness**	
Number of patients	12 (85.7%)
Mean age at diagnosis (years)	12.4 ± 5.4
Age range at diagnosis (min–max years)	6–25
**Other clinical findings**	
Urology defects	3 (21.4%)
Neuropsychiatric disorders	5 (35.7%)
Hashimoto’s thyroiditis	3 (21.4%)
Hypergonadotropic hypogonadism	1 (7.1%)
Hypogonadotropic hypogonadism	1 (7.1%)
Bilateral cataract	2 (14.3%)
Congenital heart defect	2 (14.3%)

## Data Availability

Not applicable.
